# Methyl-^11^C-L-methionine positron emission tomography for radiotherapy planning for recurrent malignant glioma

**DOI:** 10.1007/s12149-024-01901-z

**Published:** 2024-02-14

**Authors:** Hikaru Niitsu, Nobuyoshi Fukumitsu, Keiichi Tanaka, Masashi Mizumoto, Kei Nakai, Masahide Matsuda, Eiichi Ishikawa, Kentaro Hatano, Tsuyoshi Hashimoto, Satoshi Kamizawa, Hideyuki Sakurai

**Affiliations:** 1 Department of Radiation Oncology and Proton Medical Research Center, Faculty of Medicine, University of Tsukuba, 2-1-1 Amakubo, Tsukuba, Ibaraki 305-8576 Japan; 2 Department of Radiation Oncology, Kobe Proton Center, 1-6-8, Minatoshima-Minamimachi, Kobe, 650-0047 Japan; 3 Department of Neurosurgery, Faculty of Medicine, University of Tsukuba, 1-1-1 Tennodai, Tsukuba, Ibaraki 305-8575 Japan; 4 Department of Applied Molecular Imaging, Faculty of Medicine, University of Tsukuba, 1-1-1 Tennodai, Tsukuba, Ibaraki 305-8575 Japan; 5 Department of Radiology, AIC Imaging Center, 2-1-16 Amakubo, Tsukuba, Ibaraki 305-0005 Japan

**Keywords:** ^11^C-methionine, Positron emission tomography, Glioma, Radiotherapy planning

## Abstract

**Objective:**

To investigate differences in uptake regions between methyl-^11^C-L-methionine positron emission tomography (^11^C-MET PET) and gadolinium (Gd)-enhanced magnetic resonance imaging (MRI), and their impact on dose distribution, including changing of the threshold for tumor boundaries.

**Methods:**

Twenty consecutive patients with grade 3 or 4 glioma who had recurrence after postoperative radiotherapy (RT) between April 2016 and October 2017 were examined. The study was performed using simulation with the assumption that all patients received RT. The clinical target volume (CTV) was contoured using the Gd-enhanced region (CTV(Gd)), the tumor/normal tissue (T/N) ratios of ^11^C-MET PET of 1.3 and 2.0 (CTV (T/N 1.3), CTV (T/N 2.0)), and the PET-edge method (CTV(P-E)) for stereotactic RT planning. Differences among CTVs were evaluated. The brain dose at each CTV and the dose at each CTV defined by ^11^C-MET PET using MRI as the reference were evaluated.

**Results:**

The Jaccard index (JI) for concordance of CTV (Gd) with CTVs using ^11^C-MET PET was highest for CTV (T/N 2.0), with a value of 0.7. In a comparison of pixel values of MRI and PET, the correlation coefficient for cases with higher JI was significantly greater than that for lower JI cases (0.37 vs. 0.20, *P* = 0.007). D50% of the brain in RT planning using each CTV differed significantly (*P* = 0.03) and that using CTV (T/N 1.3) were higher than with use of CTV (Gd). V90% and V95% for each CTV differed in a simulation study for actual treatment using CTV (Gd) (*P* = 1.0 × 10^–7^ and 3.0 × 10^–9^, respectively) and those using CTV (T/N 1.3) and CTV (P-E) were lower than with CTV (Gd).

**Conclusions:**

The region of ^11^C-MET accumulation is not necessarily consistent with and larger than the Gd-enhanced region. A change of the tumor boundary using ^11^C-MET PET can cause significant changes in doses to the brain and the CTV.

**Supplementary Information:**

The online version contains supplementary material available at 10.1007/s12149-024-01901-z.

## Introduction

Gliomas comprise 20% of primary brain tumors in Japan. The 2016 World Health Organization (WHO) classification of gliomas includes grades 1 to 4 with malignant glioma classified as grades 3 and 4. Grade 3 [anaplastic astrocytoma (AA) and anaplastic oligodendroglioma (AO)] and grade 4 [glioblastoma (GBM)] cases have 5-year overall survival (OS) rates of 41.1% for AA, 67.8–68.7% for AO, and 10.1% for GBM [1].

Tumor resection is the main treatment for malignant gliomas, with postoperative radiotherapy (RT) and chemotherapy, but many cases have recurrence at the primary site. Re-irradiation, systemic therapy, and best supportive care are used for recurrent glioma, but outcomes are poor, with median progression-free survival after re-irradiation of 6–13 months and median OS of about 9.7–17 months [2, 3].

Re-irradiation may be the final possible treatment for glioma, and thus, a sufficient dose must be administered to suppress the tumor as much as possible, while the dose to normal brain tissue should be reduced to minimize brain damage. Accurate tumor contouring is very important to achieve these goals with greater precision. In treatment using RT, targets are determined based on the contrast-enhanced area or high signal region on T2-weighted image (WI) on magnetic resonance imaging (MRI) [4–6]. It is thought contrast-enhanced area is associated with blood–brain barrier (BBB) disruption and high signal region on T2WI includes edematous changes to these areas. However, the structure within the tumor is complex, with a mixture of active tumor cells, necrotic, and fibroblastic cells. The accuracy for determining the target volume is uncertain because it is unclear if MRI findings truly reflect the boundary of the active tumor tissue and difficult to distinguish between recurrence, pseudo progression and radiation necrosis.

Methyl-^11^C-L-methionine (^11^C-MET) is a positron emission tomography (PET) tracer that reflects intracellular amino acid metabolism [7]. MET is transported into cells and across the BBB by L-type amino acid transporters, and induction of transporters by tumor cells increases MET accumulation [8, 9]. The sensitivity and specificity of ^11^C-MET PET for brain tumors is 76–100 and 75–100%, respectively [9–15]. Glaudemans et al. suggested that ^11^C-MET PET is helpful for outlining the tumor volume and is more accurate than computed tomography (CT) and MRI in a review article based on several papers [9, 16–20]. However, a variety of methods exist to evaluate ^11^C-MET PET, and there is no widely accepted common method such as the standard uptake values for ^18^F-fluoro-2-deoxy-2-D-glucose (FDG) PET. One relatively commonly used method is the tumor-to-normal brain radioactive number ratio (T/N ratio). Appropriate T/N ratios have been proposed based on pathological findings [13, 21], MRI [22, 23], and treatment course [8, 24–26], etc. T/N ratios have generally been clinically applied between 1.3 and 2.0 in past reports.

^11^C-MET PET is likely to be useful for RT planning for malignant gliomas and has been implemented in some clinical settings. This method is often used to determine the indication of the treatment and extent of the lesions. However, several clinical issues remained unsolved. First, it is unclear to what extent MRI and ^11^C-MET PET differ for tumor extension. Second, it is unclear whether MRI or ^11^C-MET PET more accurately represents tumor extension when there is a large discrepancy in the tumor contour. Third, there are no clear criteria for establishing the tumor boundary zone on ^11^C-MET PET, and it is uncertain how establishment of this zone may influence the effectiveness of treatment. Although both are used as imaging modalities for tumors, different imaging findings are to be expected because the mechanisms of drug delivery are very different. The purpose of this study is to compare the tumor size and shape when planning treatment with MRI and ^11^C-MET PET, and to examine the possible impact of MRI and ^11^C-MET PET, plus setting of the tumor boundary region in ^11^C-MET PET, on treatment efficacy.

## Materials and methods

This study was a single-center study. This study was approved by the institutional review board of our hospital (number R03-142) and was performed in accordance with the Declaration of Helsinki and the ethical guidelines for epidemiologic research developed by the Ministry of Education, Culture, Sports, Science and Technology and the Ministry of Health, Labour and Welfare in Japan. In view of the retrospective study design, we obtained patient consent via the opt-out method using the hospital’s website.

### Patients

Twenty consecutive patients with grade 3 or 4 glioma who had recurrence after postoperative RT and underwent ^11^C-MET PET between April 2016 and October 2017 were investigated in the study (men: 17; women: 3; age: 30–72 years old). The 2016 WHO classification was grade 3 in 13 cases and grade 4 in 7 cases. Informed consent was obtained for all patients to study using ^11^C-MET PET scan images taken for diagnostic purposes. Definition of recurrence in our facility is longitudinally increased Gd enhanced area over serial MRI examinations. Moreover, high signal intensity on diffusion-weighted images and mixed or hypointense on apparent diffusion coefficient (ADC) maps were also used as a diagnostic reference. Radiation necrosis was diagnosed by the findings of T1/T2 mismatch (lack of distinct lesion margins on T2WI similar to Gd-enhanced margins on T1WI) [27], high ADC values, and low Cho on MR spectroscopy. The clinical course and abnormal accumulation of ^11^C-MET PET are used to help diagnose recurrence. Only in cases where MRI was difficult to determine, the absence of abnormal accumulation on ^11^C-MET PET was used to diagnose necrosis. In this study, all patients showed longitudinally increased Gd enhanced area. A total of 12 patients received ^11^C-MET PET to make the diagnosis of recurrence more accurate. The remaining 8 patients received ^11^C-MET PET to differentiate the brain necrosis. In practice, 5 patients received reirradiation with a prescription dose of 30–60 Gray (Gy) using conventional RT [28] and 6 patients were treated with surgery. After retreatment, all patients who underwent surgery had pathologically proven recurrence. The remaining patients, including those who chose best supportive care, were confirmed to have recurrence from subsequent follow-up. Definition of re-recurrence after post treatment was done in the same way.

### MRI

MRI was performed with a 1.5 or 3.0 T instrument (Achieva; Philips, Best, The Netherlands). T1WI after intravenous administration of 0.2 mL/kg Gd contrast agent were taken at a slice thickness of 0.9 mm.

### ^11^C-MET PET

A total of 370 (±10%) MBq of ^11^C-MET was injected intravenously at a rate of 1 mL/min, followed by flushing the infusion line with normal saline. A Biograph 16 Truepoint TV PET/CT scanner (Siemens Healthcare, Erlangen, Germany) was used for brain imaging. Data acquisition began with attenuation correction CT using a standard protocol of 120 keV and 250 mAs. This was followed by PET with a 10 min emission time per field of view. Image reconstruction was performed with the standard ordered-subset expectation maximization (OSEM) method using 24 subsets and 3 iterations with a 336 × 336 matrix using Biograph 16 Truepoint TV.

### Target delineation and RT planning with analysis

Fusion of MRI and ^11^C-MET PET images was performed using CT as a reference for RT planning aid system using MIM (MIM Software Inc., Cleveland, OH). CTV was regarded as the same as GTV and contoured using MRI and ^11^C-MET PET, respectively. For the CTV using MRI, the Gd-enhanced region was applied (CTV (Gd)). For the CTV using ^11^C-MET PET, a T/N ratio of ^11^C-MET of 1.3 (CTV (T/N 1.3)), a T/N ratio of 2.0 (CTV (T/N 2.0)), and the PET-edge method in MIM (CTV(P-E)) were applied. Threshold values for ^11^C-MET accumulation have been proposed based on a variety of evidence [8, 13, 21–26]. Among these, we selected T/N 1.3 and 2.0, considering the balance between low and high settings. The normal area count was calculated from the counts of several regions of interest placed on the opposite side of the brain. We also used the PET edge method. The PET-edge method calculates the spatial derivative along the tumor radius and defines the tumor edge based on the derivative level and the continuity of the tumor edge [29]. Contouring was performed in consultation with two radiation oncologists (25 and 8 years of experience).

Gd-enhanced and ^11^C-MET accumulation regions were examined using two methods.

First, the concordance rates of CTV (Gd) with CTV (T/N 1.3), CTV (T/N 2.0), and CTV (P-E) were calculated using the Dice Score Coefficient (DSC) and Jaccard Index (JI):

$${\text{DSC}} = \frac{{2\left| {M \cap N} \right|}}{\left| M \right| + \left| N \right|}\quad {\text{JI = }}\frac{{\left| {M \cap N} \right|}}{{\left| {M \cup N} \right|}},$$

where *M* and *N* represent each CTV.

Second, the distributions of Gd and ^11^C-MET were examined by comparing pixel values from Gd-enhanced MRI and ^11^C-MET PET.

RayStation (RaySearch Laboratories., Stockholm, Sweden) was used for RT planning. Comparison of each CTV and two simulation studies were performed using stereotactic radiotherapy (SRT) with the conformal arc technique with 39 Gy/13 fr. The conformal arc technique used 6 arc beams with a leaf margin of 2.0 mm. The planning target volume (PTV) average dose was used as the prescription dose, the same method used in SRT with a conformal arc at the time several of the patients in this subject were actually treated.

First, changes of dose distribution to normal brain were simulated with different CTVs, with the distribution calculated using the four types of CTV. To analyze the brain dose, the brain D50% (absolute dose received by 50% of the volume), D1% and Dmax were examined. Second, the dose distributions for CTV (T/N 1.3), CTV (T/N 2.0), and CTV (P-E) were examined based on the actual treatment using CTV (Gd). The V90% (percentage of the target receiving 90% of the prescribed dose) and V95% of the CTV were calculated as indices to evaluate the dose to the CTV as defined by ^11^C-MET PET.

A single-factor ANOVA with Bonferroni correction or an unpaired *t *test was used to compare data among the groups. Pearson correlation coefficients (*r*) were used in comparison of pixel values. A *P* value <0.05 was considered to indicate significance in all analyses.

## Results

### Comparison of Gd-enhanced and ^11^C-MET accumulation regions

**Fig. 1:**
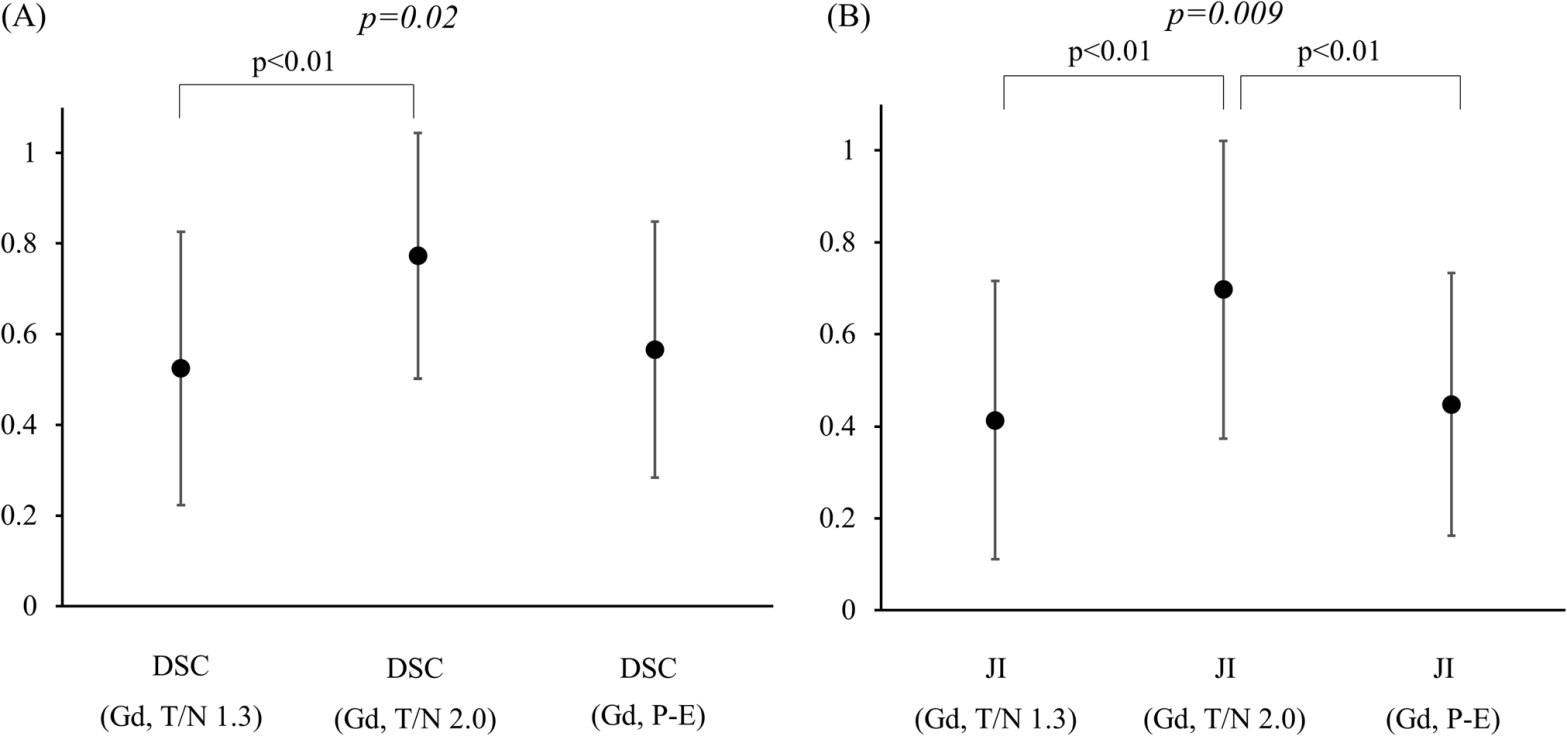
Concordance rate between CTV (Gd) and CTV (T/N 1.3), CTV(T/N 2.0), CTV(P-E). **a** Dice Score Coefficient (DSC). **b** Jaccard Index (JI). *P* value for single-factor ANOVA analysis is shown in *italicized font* and Bonferroni correction analysis is shown in regular font

**Fig. 2:**
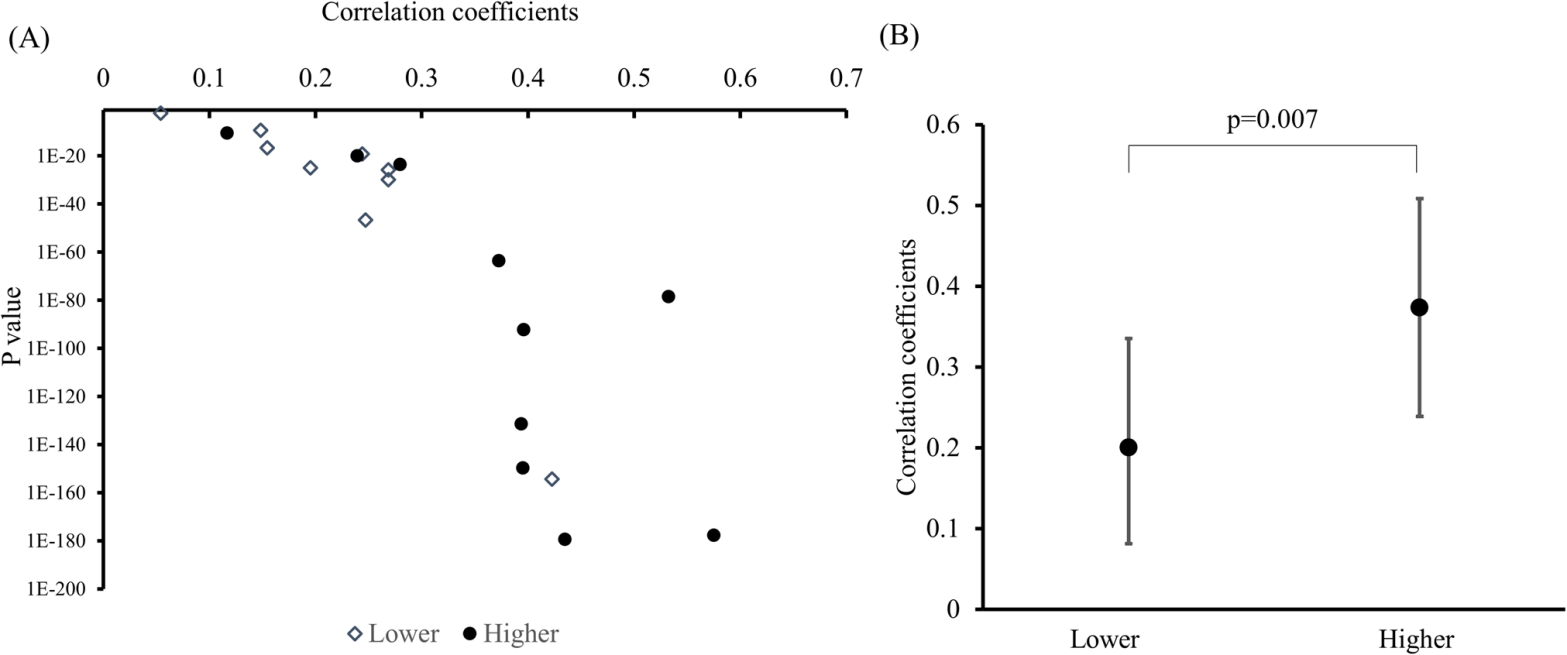
**a** Correlation coefficient and *P *value for mapping of pixel values between MRI and ^11^C-MET PET. **b** Correlation coefficient in patients with higher and lower JI

CTV data are shown in Table 1. In comparison with CTV (Gd), DSCs differed significantly among CTVs (*P* = 0.02, single-factor ANOVA), with the DSC of CTV (Gd) and CTV (T/N 2.0) (DSC (Gd, T/N 2.0)) being higher than DSC (Gd, T/N 1.3). There were similar significant differences in JI (*P* = 0.009), and JI (Gd, T/N 2.0) was higher than JI (Gd, T/N 1.3) and JI (Gd, P-E) (Fig. 1a and b). The correlation coefficients (*r*) for pixel values from Gd-enhanced MRI and ^11^C-MET PET were <0.3 in 9/10 patients with a lower JI, but >0.3 in 7/10 patients with a higher JI. The *r* of higher JI patients was 0.37, which was significantly stronger that of lower JI patients (*r* = 0.20), (*P* = 0.007) (Fig. 2a and b).

**Table 1:** CTV data

Parameter	CTV (Gd)	CTV (T/N 1.3)	CTV (T/N 2.0)	CTV (P-E)
Volume (cm^3^)	7.6–24.8 (16.2 ± 8.6)	18.8–68.4 (43.6 ± 24.8)	10.8–28.5 (19.6 ± 8.9)	13.4–62.2 (37.8 ± 24.4)
Overlap volume with CTV (Gd) (cm^3^)		7.2–23.5 (15.3 ± 8.2)	6.0–21.6 (13.8 ± 7.8)	7.0–23.5 (15.2 ± 8.2)
Dice Score Coefficient (DSC)		0.03–0.96 (0.52 ± 0.3)	0.09–1.0 (0.77 ± 0.27)	0.15–0.96 (0.57 ± 0.28)
Jaccard Index (JI)		0.01–0.93 (0.41 ± 0.3)	0.05–1.00 (0.70 ± 0.32)	0.08–0.92 (0.48 ± 0.29)

Numbers in parentheses show the minimum–maximum and the mean ± standard deviation

*CTV* clinical target volume, *T/N* tumor/normal tissue ratio, *DSC* dice score coefficient, *JI* Jaccard Index

### Simulation study

**Fig. 3:**
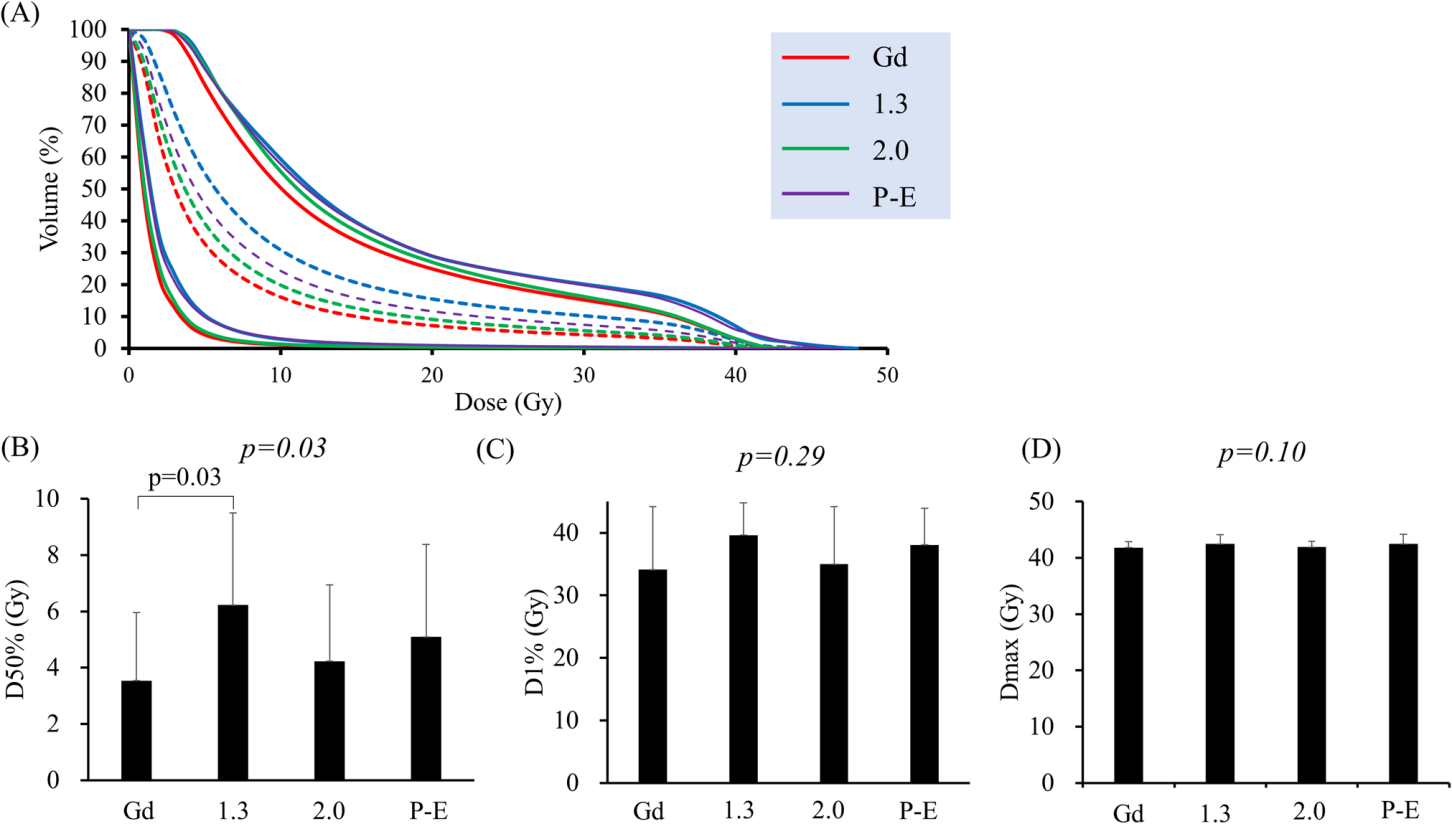
Dose-volume data for the brain. **a** Dose volume histogram. Solid lines show the maximum and minimum, and the *dotted line* is the average. **b** D50%. **c** D1%. **d** Dmax. D50% means dose of 50% volume of the target (unit: Gy). Data are shown as means and standard deviations. *Red*: CTV(Gd), *blue*: CTV (T/N 1.3), *green*: CTV (T/N 2.0), *purple*: CTV (P-E). *P* value for single-factor ANOVA analysis is shown in *italicized font* and Bonferroni correction analysis is shown in regular font (color figure online)

D50% of the brain was 3.5 ± 2.4 Gy using CTV (Gd), 6.2 ± 3.3 Gy at CTV (T/N 1.3), 4.2 ± 2.7 Gy at CTV (T/N 2.0), and 5.1 ± 3.3 Gy at CTV (P-E), respectively. D1% was 34.1 ± 2.2, 39.6 ± 1.2, 35.0 ± 2.1, and 38.1 ± 1.3 Gy, respectively. Dmax was 41.8 ± 0.24, 42.5 ± 0.36, 41.9 ± 0.23, and 42.5 ± 0.38 Gy, respectively. There were significant differences in D50% among CTVs (*P* = 0.03, single-factor ANOVA) and these values with CTV (T/N 1.3) were higher than that of CTV (Gd). There were no significant differences in D1% and Dmax among CTVs (*P* = 0.29 and 0.10, respectively, single-factor ANOVA) (Fig. 3).

**Fig. 4:**
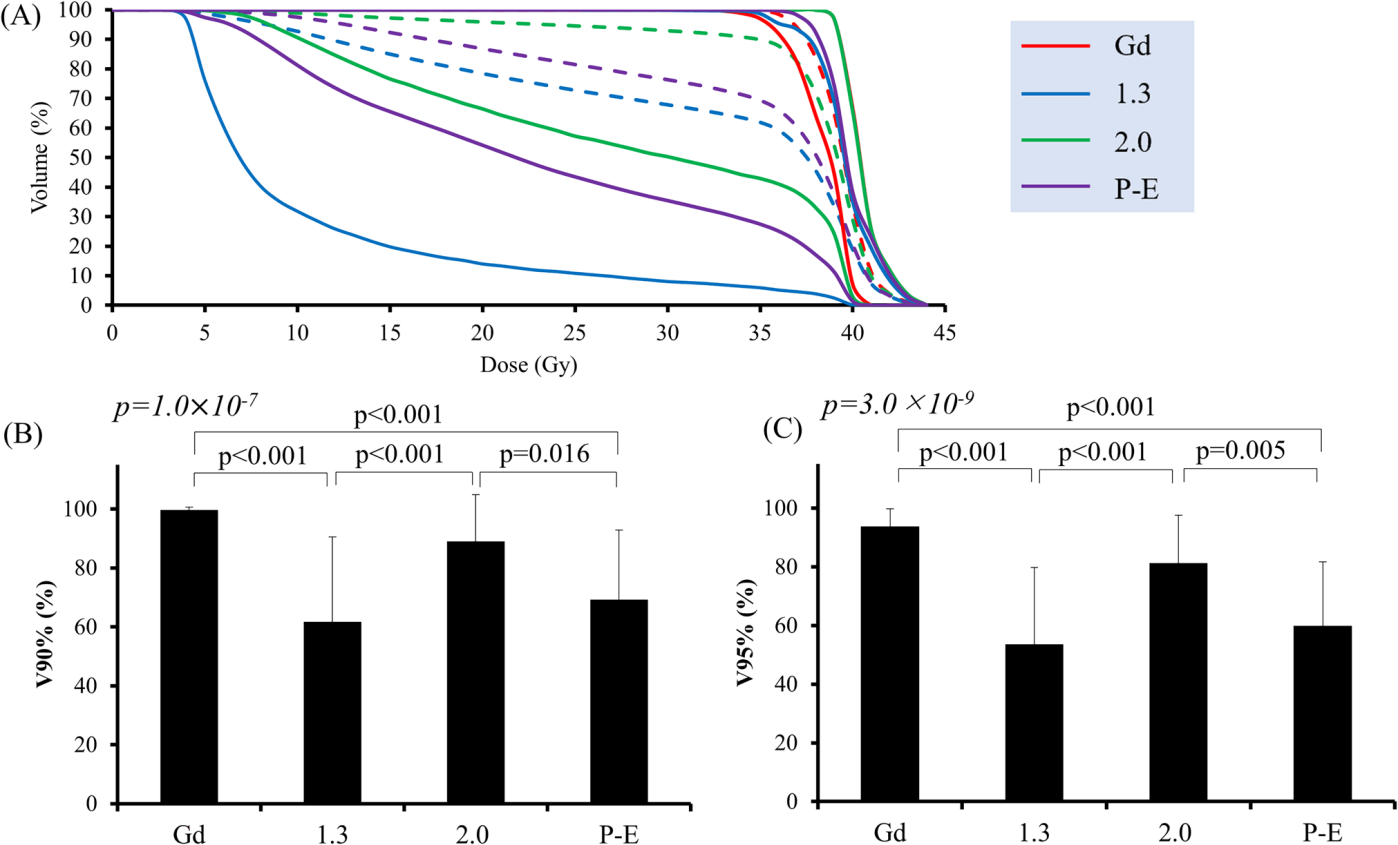
Dose-volume data for each CTV. Data represent each CTV dose in actual treatment using CTV (Gd). **a** Dose volume histogram. **b** V90%. (C) V95%. The notation of the graphs is the same as that in Fig. 3. *P* value for single-factor ANOVA analysis is shown in *italicized font* and Bonferroni correction analysis is shown in regular font

V90% of the CTV was 99.6 ± 1.0% using CTV (Gd), 61.7 ± 28.8% at CTV (T/N 1.3), 89.0 ± 15.8% at CTV (T/N 2.0), and 69.2 ± 23.6% at CTV (P-E), respectively. The V95% values were 93.7 ± 6.1, 53.5 ± 26.3, 81.2 ± 16.4, and 59.8 ± 21.9%, respectively. There were significant differences in V90% and V95% among CTVs (*P* = 1.0 × 10^–7^ and 3.0 × 10^–9^, respectively, single-factor ANOVA) and these values for CTV (T/N 1.3) and CTV (P-E) were lower than those for CTV (Gd) and CTV (T/N 2.0) as shown in Fig. 4.

**Fig. 5:**
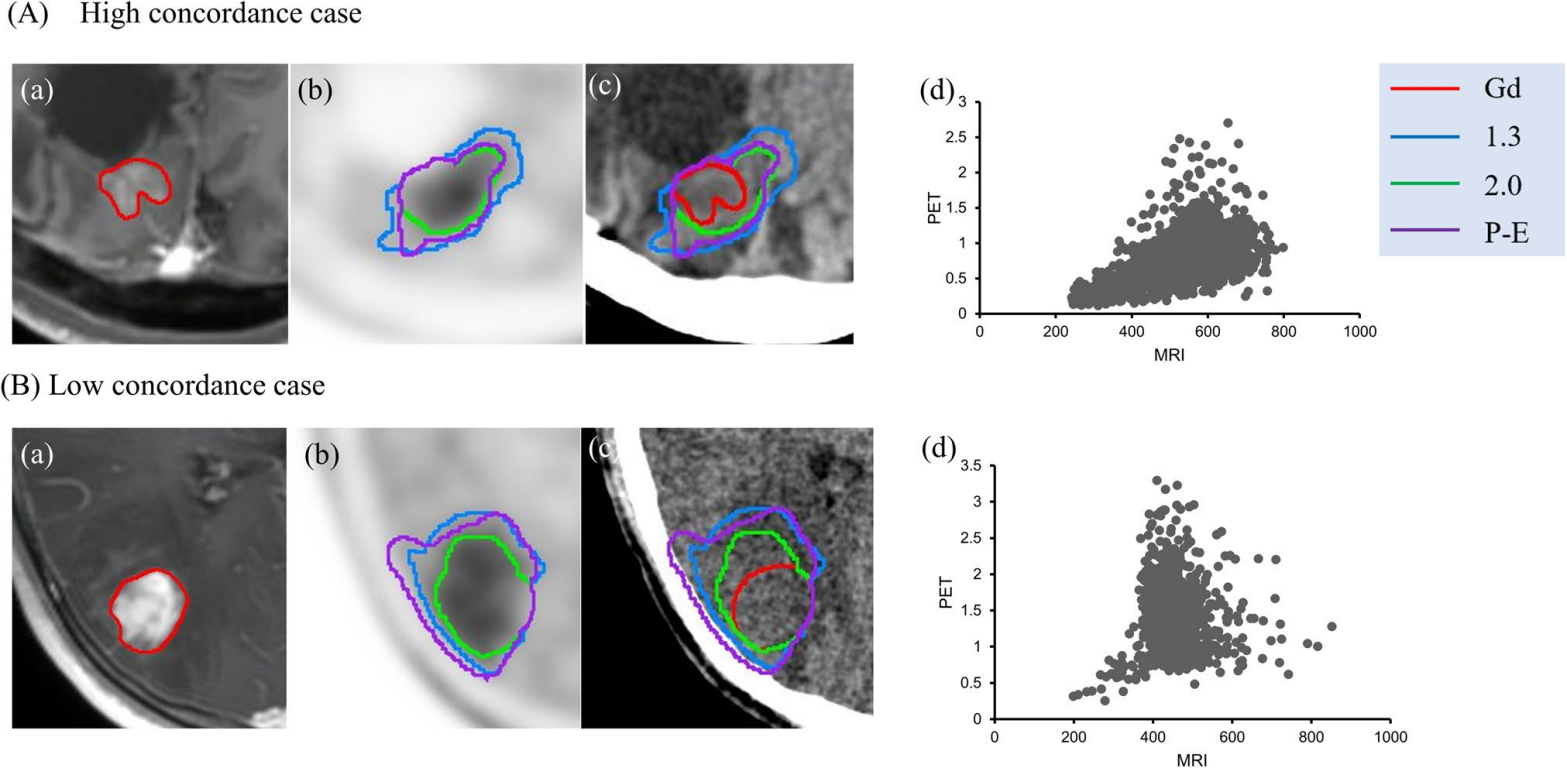
Examples of high and low concordance between Gd-enhanced and ^11^C-MET accumulation regions. **A** A 51-year-old man with a Grade 3 tumor. **B** A 36-year-old woman with a Grade 4 tumor. *a* Gd-enhanced MRI (*red*: CTV (Gd)). *b* ^11^C-MET PET (*blue*: CTV (T/N 1.3), *green*: CTV (T/N 2.0), *purple*: CTV (P-E)). *c* CTV contour overlaid on treatment-planning CT. *d* Correlation coefficient for pixel values in MRI and PET (color figure online)

Examples of high and low concordance of Gd-enhanced and ^11^C-MET accumulation regions are shown in Fig. 5. In a 51-year-old man with a Grade 3 tumor (Fig. 5a), DSC (Gd, T/N 1.3), (Gd, T/N 2.0), (Gd, P-E) were 0.41, 0.77, 0.62, respectively; and JI (Gd, T/N 1.3), (Gd, T/N 2.0), (Gd, P-E) were 0.26, 0.63, 0.46, respectively; with *r* = 0.58 for the pixel values from MRI and PET. In a 36-year-old woman with a Grade 4 tumor (Fig. 5b), DSC (Gd, T/N 1.3), (Gd, T/N 2.0), (Gd, P-E) were 0.37, 0.85, 0.41, respectively; and JI (Gd, T/N 1.3), (Gd, T/N 2.0), (Gd, P-E) were 0.23, 0.74, 0.26, respectively; with *r* = 0.004 for the comparison of MRI and PET pixels.

## Discussion

Grosu et al. found mean target volumes of 11 cm^3^ for the Gd-enhanced region and 19 cm^3^ for the ^11^C-MET region, with an overlap volume of 6 cm^3^ at a T/N ratio of 1.7. ^11^C-MET accumulation extended beyond the Gd-enhanced region in 29/39 cases (74%) [24]. In the current study, the ^11^C-MET region was larger than the Gd-enhanced region in 16/20 cases (80%) using a tumor boundary at a T/N ratio of 2.0. The mean target volumes of CTV (Gd) and CTV (T/N 2.0) were 16.2 and 19.8 cm^3^, with an overlap of 13.8 cm^3^, which is similar to the previous report. As shown in the results, the best index of overlap was 0.77 for DSC (Gd, T/N 2.0) and 0.7 for JI (Gd, T/N 2.0). However, the DSC and JI values of 0.77 and 0.7 are still not sufficiently high to indicate a good match, and some patients have values of <0.5 (DSC: 3 cases, JI: 7 cases), which indicates a weak correlation between the Gd-enhanced and ^11^C-MET accumulation regions.

In mapping of *r* and *P* values of pixel values from Gd-enhanced MRI and ^11^C-MET PET, the *r* varied from 0.004 to 0.58. The pattern seemed to fall into two groups of relatively weakly related (*r*: 0.004–0.28, *P *values: 0.87–1.75E^−47^) and relatively strongly related (*r*: 0.39–0.58, *P *values: 2.6E^−64^–2.9E^−180^) cases. There were 3 cases with higher JI and 9 cases with lower JI with *r* < 0.3, but 7 cases with higher JI and 1 case with lower JI with *r* > 0.3. The cases with high concordance between Gd-enhanced MRI and ^11^C-MET PET indicate that the BBB is disrupted in the vicinity of regions of active amino acid metabolism. However, this trend was not seen in about half of the cases, indicating that they are not necessarily in close proximity.

Pirotte et al. found that the ^11^C-MET PET volume did not match the MRI volume and improved the tumor volume delineation in 88% of low-grade glioma and 78% of high-grade glioma cases compared with surgical findings [30]. In studies using RT, Navarria et al. showed that the ^11^C-MET region was within the FLAIR high signal region plus 1 cm in all cases at a T/N ratio of 1.5, and that the ^11^C-MET region coincided with sites of recurrence [31]. Lee et al. reported recurrence in 2 of 14 patients and suggested that the ^11^C-MET area had a high risk for recurrence using a T/N ratio of 1.5 [32]. In a review, Galldiks et al. reported that ^11^C-MET regions were larger than Gd-enhanced regions and suggested that biologically active disease might extend considerably beyond the Gd enhancement area [33]. Additionally, it was suggested that ^11^C-MET could identify areas at highest risk for glioma recurrence following RT. Of the 20 patients in the current study, 5 underwent reirradiation, of whom 3 had a decrease in Gd-enhanced tumors and 2 had marginal recurrence. Use of ^11^C-MET may reduce the risk of recurrence because it includes surrounding active lesions that are not shown in Gd enhancement. However, in the 2 recurrence cases, the sites of recurrence were outside the ^11^C-MET PET boundary. The small number of cases prevents a clear answer, but establishment of reasonable boundaries remains as an issue.

The brain has a feature as a serial organ and the other as a parallel organ. The typical adverse event as a serial organ is brain necrosis, which is a definitive effect and should be evaluated at the maximum dose. On the other hand, a typical adverse event as a parallel organ is brain dysfunction. Thus, we evaluated both maximum and average brain doses to assessment in this study. In our simulation study, there was a large variation in brain dose depending on CTV settings. If CTV was determined using the Gd-enhanced region, D50% for the brain was 3.5 Gy, but this value was 6.2 Gy using T/N 1.3 of ^11^C-MET, 4.2 Gy using T/N 2.0, and 5.1 Gy using P-E. Thus, in RT planning using ^11^C-MET findings, the normal brain dose is higher than with Gd-enhanced findings, about twice as high. On the other hand, Dmax and D1% did not differ between plans using Gd and ^11^C-MET because the maximal dose could not be reduced due to the brain being in contact with the tumor. The findings from this simulation are clinically very important, but there are issues that need to be kept in mind when applying them clinically. In actual treatment, margin settings, fractionation and parameter settings should be changed depending on the tumor size and localization. Moreover, initial treatment dose must be considered carefully. However, there was no significant difference in tumor size (CTV (Gd): 7.6–24.8 cm^3^) and since this was a simulation study, the treatment plans were designed identically to exclude clinical bias and simplify comparison.

In our simulation study that evaluated the dose of each CTV in a treatment targeting the actual Gd-enhanced region, V95% of CTV (Gd) was 93.7%, 53.5% using T/N 1.3 of ^11^C-MET PET, 81.2% using T/N 2.0, and 59.8% using P-E. Even if the tumor contour was determined using T/N 2.0, which had the highest correlation with Gd, the V95% of CTV (T/N 2.0) is only about 80%. This is not sufficient for tumor control. Assuming the tumor extends to the ^11^C-MET accumulation site, this means that a treatment plan contoured by conventional MRI would result in an inadequate dose. The simulation results using other parameters showed even lower coverage of the CTV.

Although previous studies have used different tumor boundary settings and an optimal threshold value for the tumor boundary has yet to be established, some physicians have started to use ^11^C-MET PET for treatment planning. Grosu et al. found an increased mean survival period from 5 to 9 months when ^11^C-MET PET or ^123^I-α-methyltyrosine single-photon emission CT was added in treatment planning that was previously performed using contrast-enhanced MRI alone [34]. Miwa et al. reported 6-month and 1-year survival rates of 71.4 and 38.1% for recurrent lesions treated with tumor contouring with a T/N ratio of 1.3 and SRT of 25 Gy/5 fr to 35 Gy/7 fr [35]. We believe that a future challenge is to change the setting range of the tumor boundary according to the treatment strategy. For example, when the antitumor effect is to be enhanced, the T/N ratio should be set low so that there is no shortage of tumor dose. Conversely, when the effect on the normal brain is to be minimized, the T/N ratio should be set high and irradiation should be targeted to the core of the tumor to minimize the dose to the brain. Appropriate irradiation should thus be performed with a balance of treatment objectives. This is an issue that requires further consideration based on clinical evaluation.

There are some discussions which modality is more appropriate to contour malignant brain tumors. The Japanese Society for Radiation Oncology guideline states that MRI and CT should be used for contouring [36]. American Society for Radiation Oncology (ASTRO) guideline and European Society for Radiotherapy and Oncology (ESTRO) guideline state that MRI is the basic method [37, 38]. Therefore, radiation oncologists should give priority at this time to MRI over ^11^C-MET PET for contouring the target. While, some argue that amino acid PET, such as ^11^C-MET PET can cover the tumor region [32, 35]. These reports are mostly case reports or investigations for a small number of patients. However, some guidelines mention the possibility that ^11^C-MET PET can compensate for the weakness of MRI and further discussion is needed [37–40]. Although this study alone cannot build evidence to change existing guidelines, we hope that in the near future this and similar studies will contribute to a high evidence-based contribution of ^11^C-MET PET to target contouring in RT planning.

There are several limitations in this study. The mechanism and correctness of differences between ^11^C-MET and MRI remain unknown. In 6 cases, surgery was performed, and intraoperative MRI findings and 5-aminolevulinic acid-positive lesions were used to determine surgical margins. Because all suspected areas on MRI and ^11^C-MET PET were resected, and because this is a retrospective study, it is difficult to assess which modality is more appropriate for defining targets, or the association between ^11^C-MET accumulation sites and sites of post treatment re-recurrence. In order to establish more accurate contouring techniques, prospective studies such as mapping and comparing suspicious areas by MRI and PET at the time of surgery are needed. Another limitation is that treatment planning methods, including parameter setting, have developed rapidly and become more detailed in recent years, and the parameter settings used in this study are relatively less restrictive than our current protocols. In fact, at that time, only the Dmean of PTV was used at our institution as the prescribed dose for SRT using the conformal arc method, and not the D95 of PTV, etc., as is used today. This study was conducted to compare the dose distribution when CTV was different from the actual treatment. Therefore, for the simulations in this study, we decided to use the parameter settings that were used in actual treatment in the mid-2010s.

We expect the results of this study will help to address this fundamental issue and establish the significance and effectiveness of using ^11^C-MET PET for contouring in RT planning.

## Conclusion

The CTV using ^11^C-MET PET is larger than that using the Gd contrast area, and the overlap is at best estimated to be about 70%. A high match rate cannot be expected only by adjusting the threshold value and the region of ^11^C-MET accumulation is not necessarily similar to the Gd-enhanced region. A change of the tumor boundary zone based on the threshold value of ^11^C-MET PET causes significant changes in the brain and CTV doses. Careful tumor boundary setting is important and a more detailed discussion of this approach is needed for safe and effective RT for brain tumors.

### Supplementary Information

Below is the link to the electronic supplementary material.

Supplementary material 1 (XLSX 495 KB)

## Data Availability

The authors confirm that the data supporting the findings of this study are available within the article and its supplementary materials.
